# Mastering Therapeutic Drug Monitoring: Essential Strategies for Pregnancy and Breastfeeding

**DOI:** 10.1192/j.eurpsy.2025.264

**Published:** 2025-08-26

**Authors:** G. Schoretsanitis

**Affiliations:** Department of Psychiatry, Psychotherapy and Psychosomatics, Hospital of Psychiatry, Zurich, Switzerland

## Abstract

**Abstract:**

Measuring medication levels in blood and/or milk of psychotropic-treated women during pregnancy and lactation provides significant benefits for optimizing maternal mental health while minimizing risks to the developing fetus or breastfeeding infant. This approach supports individualized treatment plans by addressing the unique pharmacokinetic and pharmacodynamic changes that occur during these physiological states. Among key benefits monitoring blood levels ensures that psychotropic medications remain within therapeutic ranges, thereby reducing the likelihood of relapse while avoiding toxicity. Moreover, measuring drug levels aids in balancing maternal benefits against fetal risks by enabling dose adjustments to minimize unnecessary fetal exposure while maintaining efficacy. It is particularly relevant for medications with narrow therapeutic indices or significant placental transfer. Additionally, pregnancy induces changes in drug absorption, distribution, metabolism, and excretion, which can lead to subtherapeutic levels. Monitoring essentially helps clinicians anticipate and adjust for these alterations, ensuring consistent drug efficacy. Further, postpartum, measuring drug levels in maternal blood and, when appropriate, in breastmilk provides data on infant exposure risks. This information is crucial for determining the safety of breastfeeding while continuing psychotropic therapy.

Last, drug level data allow clinicians to assess adherence, which is a major aspect during pregnancy and lactation.

Incorporating medication level assessments into risk management frameworks during pregnancy and lactation ensures that women receive evidence-based, safe, and effective psychotropic treatment while supporting fetal and neonatal well-being.

**Disclosure of Interest:**

None Declared

